# The role of microglia in the development of diabetic retinopathy

**DOI:** 10.1038/s44324-024-00009-2

**Published:** 2024-06-03

**Authors:** Pialuisa Quiriconi, Vanco Hristov, Mayu Aburaya, Una Greferath, Andrew I. Jobling, Erica L. Fletcher

**Affiliations:** Department of Anatomy and Physiology, The University of Melbourne, Melbourne, VIC Australia

**Keywords:** Endocrinology, Endocrine system and metabolic diseases

## Abstract

Diabetic retinopathy is a vision-threatening disease and remains the most feared complication for those living with diabetes. Historically, the disease has been considered primarily vascular in nature, based on clinically detectable vascular pathology. Nonetheless, it is now recognized that the retina undergoes a variety of cellular changes from the early onset of diabetes. In fact, one of the earliest changes to occur is a loss in vasoregulation, yet our understanding of the underlying mechanisms is lacking. Microglia, the resident immune cells of the central nervous system, perform a range of physiological, non-inflammatory functions to maintain retinal homeostasis which includes surveying the microenvironment to constantly monitor tissue health, neuronal surveillance to maintain synaptic integrity and vasoregulation, a recently discovered role that these cells additionally perform. The role of microglia in the development of diabetic retinopathy is well-established, centered around their contribution to inflammation which remains an integral component in disease pathogenesis, particularly in later stages of disease. However, recent findings reveal that early in the development of diabetes the vasoregulatory function of microglia is dysfunctional, leading to early vascular compromise. This review summarizes recent work to highlight how microglia are affected by diabetes and the implications of these changes in the development of diabetic retinopathy from pre-clinical to advanced stages of disease.

## Introduction

Diabetic retinopathy is the most feared complication of diabetes mellitus, affecting approximately 22.3% of those with diabetes worldwide^1^. The prevalence of this disease is predicted to rise by 55.6% (57.4 million people) from 2020–2045^1^. This is attributed to the ongoing diabetes epidemic, predominately driven by the increase in type 2 diabetes, which accounts for ~90–95% of all diabetes cases^2,3^. Currently, treatment is only available for the late stages of the disease to prevent further visual deterioration. A deeper understanding of the underlying mechanisms in the pathogenesis of diabetic retinopathy is therefore critical for the development of novel interventions to prevent disease onset or delay progression.

Vision loss arises in the late stages of diabetic retinopathy due to neovascularization, the pathological proliferation of new blood vessels, on the surface of the retina and/or oedema within the vision sensitive macula region of the retina^4^. The presence of such visible vascular pathology forms the basis of clinical diagnosis and management of the disease. This has shaped the historic view that diabetic retinopathy is a microvascular disease. However, over the last decade there has been increasing recognition that diabetes impacts the entire retina, including retinal neurons and support cells such as glia and microglia. In fact, changes in retinal function are documented to predate the development of clinically defined diabetic retinopathy as functional abnormalities such as deficits in visual acuity, contrast sensitivity and retinal function assessed by multifocal electrical retinography, are reported to occur in those with diabetes in the absence of overt vascular pathology^5,6^. Specific sites in the diabetic retina exhibiting neuronal dysfunction are notably reported to predict the areas in which vascular pathology later manifest^7^. In addition to neuronal change, subtle changes in the regulation of blood flow are also present as one of the earliest pre-clinical changes to occur in the diabetic eye, with numerous studies revealing vasoregulatory impairments to occur early in the development of disease^8–10^. As a metabolically demanding tissue, retinal function is dependent on adequate perfusion and so even minor disruptions in blood flow can have severe consequences on retinal health and functioning.

Microglia are the resident immune cells of the central nervous system (CNS) that constantly survey the environment to assess the health status of the tissue. These cells are the frontline responders to injury and disease, but additionally perform a variety of critical homeostatic functions, with recent work revealing that this also includes vasoregulation^11,12^. In diabetic retinopathy, the most studied role of microglia in disease pathogenesis is centered around their contribution to retinal inflammation. However, anomalies in microglial-mediated vasoregulation have also recently been described^12^. In this review, we summarize recent work on the contribution of microglia at different stages of diabetic retinopathy from pre-clinical to clinical stages of disease, highlighting the therapeutic potential in targeting these cells to improve visual outcomes in diabetic retinopathy.

## Microglia – more than just resident immune cells of the CNS

**Fig. 1:**
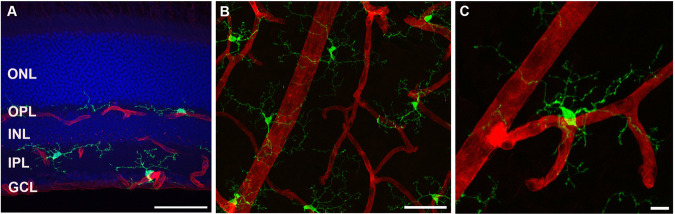
Association of microglia with the vasculature in the mouse retina. Within the retina, microglia somata are located in the ganglion cell layer (GCL), inner plexiform layer (IPL), and outer plexiform layer (OPL), with their processes extending throughout and beyond these layers. **A** Vertical section of the Cx3cr1^+/GFP^ mouse retina was stained for microglia (GFP, green), blood vessels (IB4, red), and nuclei (DAPI, blue) (**B**, **C**). En face wholemount images of the mouse retina immunolabelled for microglia (GFP, green) and blood vessels (IB4, red), depicting the stellate morphology of microglia and microglial-vascular interactions. The close association between microglia and vasculature is further illustrated at the single-cell level in image (**C**). Scale bars = 50 μm (**A**,, **B**), 10 μm (**C**).

Microglia are typically characterized as the resident innate immune cells of the CNS, including the retina^13^. They arise from the embryonic yolk sac to colonize the CNS including the retina and are long-lived cells^14,15^. As shown in Fig. 1, microglia in the mature retina are ramified cells with somata localized to three main regions, the ganglion cell layer (GCL), inner plexiform layer (IPL), and outer plexiform layer (OPL), whilst processes typically project beyond these layers to effectively tile the whole retina^16^. Through the expression of a variety of receptors that detect extracellular cues, microglia are important sensors of the health status of the retina and as immune cells, they are critical responders to retinal injury, infection or disease. Exemplifying this, microglia readily adopt an activated phenotype in response to pathological stimuli, evidenced by retraction of processes and increased soma volume (ameboid phenotype), proliferation, and migration to sites of injured or damaged tissue where they express various cytokines and chemokines^17,18^. This inflammatory reaction by microglia is thought to be protective of tissue homeostasis, by limiting the spread of damage, clearing debris, and repairing affected tissue.

It was once a commonly held view that microglia were quiescent cells awaiting activation by a pathogenic stimulus, with almost a negligible contribution to tissue homeostasis^13^. However, a growing body of literature has challenged this notion, by revealing that microglia execute many additional functions beyond a standard immune cell, that are vital for the health and normal functioning of the CNS, including the retina. In the developing visual system, microglia are involved in a range of functions including programmed cell death, clearance of apoptotic cells and synaptic pruning^11^. Thus, these cells execute important roles in establishing and refining the neural network and circuitry. Notably, microglia in the retina are also required for cone photoreceptor maturation, horizontal cell organization and normal vascular development^19–21^.

Furthermore, in the mature retina, it is now widely recognized that microglia ‘at rest’ are far from dormant and are in fact very active, evidenced by the high dynamics in microglial processes and arborization^22–24^. This allows microglia to constantly survey the microenvironment to assess the health status of parenchyma. Importantly, microglia make contact with synapses in an activity-dependent manner, allowing them to partake in neuronal surveillance, which entails monitoring neuronal stability, transmission, function as well as synaptic pruning^22,25^. This is a critical function for maintaining normal vision. Highlighting this, sustained microglial depletion in the mature mouse retina specifically resulted in the compromised structural and functional integrity of synapses, accompanied with reduced retinal function, whilst retinal architecture remained unaffected^21^.

## The retina is an energy-demanding tissue dependent on adequate vasoregulatory mechanisms that also involve microglia

The retina is one of the most energy demanding tissues within the nervous system^26,27^. Thus, blood vessels and their regulation are of critical importance in appropriately supplying the retina with the oxygen and nutrients needed, whilst removing waste products. Moreover, unlike other tissues in the body, the retina possesses limited capacity for glucose storage and so both oxygen and glucose need to be readily available and their delivery tightly controlled and tunable to metabolic demand of neurons.

**Fig. 2:**
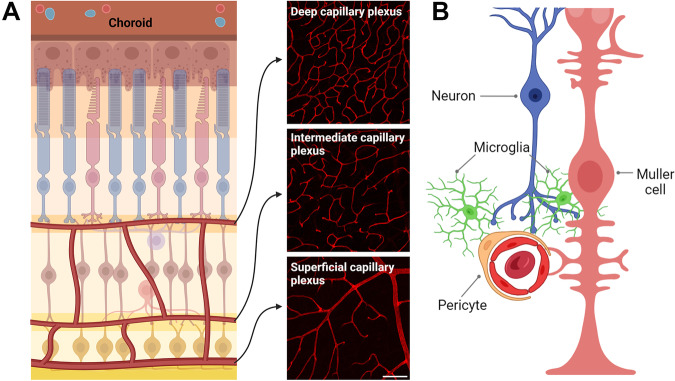
Summary of the vascular supply to the retina and the cells that interact with the vasculature. **A** Diagram of the vasculature within the mammalian retina that crucially supplies oxygen and nutrients to this energy-demanding tissue. Retinal vasculature is dual in nature, consisting of choroidal and retinal circulation. The choroidal vasculature (choroid) sits behind the retina, nourishing the outer retinal neurons. The retinal vasculature primarily supplies the inner retina through several capillary networks; the superficial, intermediate, and deep vascular plexuses (SVP, IVP, and DVP) that reside in the ganglion cell layer (GCL), inner plexiform layer (IPL), and outer plexiform layer (OPL), respectively. **B** Diagram of the neurovascular unit (NVU) that includes structural and functional connections between neurons, glia, microglia and vasculature. One of the primary functions it serves is blood flow regulation, through mediating neurovascular coupling. This enables neurons to signal directly with vasculature or indirectly through an intermediate glial/microglial cell, to modulate vessel caliber and thus blood flow appropriately in accordance with changes in neuronal activity.

Optimal functioning of the retina is dependent on adequate metabolic support provided by a dual vascular supply, retinal and choroidal circulation^28^. Retinal circulation travels through the optic nerve and branches into arterioles, forming several capillary networks (or plexuses) within the synaptic layers of the retina; the superficial vascular plexus (SVP, located within the ganglion cell layer), intermediate vascular plexus (IVP, located in the inner plexiform layer) and deep vascular plexus (DVP, located in the outer plexiform layer), depicted in Fig. 2. The SVP and IVP primarily supply the inner retinal neurons, while the DVP supplies both inner retinal neurons and a small component of photoreceptor needs. Generally, the majority of the outer retina, including the photoreceptors and retinal pigment epithelium (RPE) is supplied by the choroidal circulation, which is formed from the long and short posterior ciliary arteries^28,29^. With regard to venous drainage, the capillaries from the inner retinal circulation drain into venules surfacing at the superficial retina which exit along the optic nerve^28,30^. Choroidal drainage alternatively occurs via vortex veins which drain into the superior and inferior ophthalmic veins^31,32^. Retinal vasculature is governed by robust mechanisms to ensure a constant uninterrupted blood supply. Importantly, the dual blood supply of the retina is differentially regulated, as sympathetic innervation controls choroidal circulation, whilst retinal circulation lacks sympathetic input and is thus regulated independently by local metabolically driven mechanisms^30^. Since inner retinal vasculature responses are principally compromised in diabetic retinopathy (DR), this review will focus on the vasoregulatory mechanisms of this specific blood supply. For a review of the mechanisms governing choroidal blood flow, please refer to Kur et al.^33^

The retinal circulation is governed by several vasoregulatory mechanisms, notably autoregulation and metabolic regulation. Autoregulation ensures that retinal blood flow remains constant, under a variety of conditions including changes in systemic blood pressure, intraocular pressure, or alterations in blood oxygen levels^33^. One prominent mechanism by which this is achieved is through the myogenic response, where fluctuations in intravascular pressure are sensed by vascular smooth muscle cells surrounding arteries and arterioles, influencing their contractile state which subsequently leads to adjustments in vessel tone and blood flow^34^. Moreover, other local vasoregulatory mechanisms are additionally employed to ensure that blood flow is distributed appropriately to areas of priority at any given time, given the heterogeneity in metabolic demand across the tissue. This includes metabolic regulation, also termed neurovascular coupling (NVC), in which neuronal activity is tightly coupled to vascular responses, to regulate blood flow appropriately^35^. NVC is achieved through the coordinated response of neurons, glia, and vascular support cells (endothelial cells, vascular smooth muscle cells, and pericytes), which function in unison to correlate neuronal need with an appropriate change in blood flow^35^. This group of cells is collectively named the neurovascular unit (NVU), illustrated in Fig. 2. Evidence for this type of neuronally driven vascular response was first described over a century ago, by Mosso in humans and Roy and Sherrington in animals and is termed functional hyperaemia^36–38^.

The role of the NVC has been demonstrated in the retina, where, for example, a flickering light stimulus, evokes a ~3-5% dilation in retinal arterioles and venules^39–42^. Similar findings have been observed in the rodent retina, where vasodilatory responses alongside increases in red blood cell flux, are observed by retinal arterioles, venules as well as capillaries across all plexuses in response to flicker stimulation^43,44^. The changes in vessel caliber and associated changes in blood flow, are assumed to accommodate for alterations in metabolic requirements by neurons based on the status of their activity. NVC thus prevents any mismatch between neuronal activity and metabolic support and additionally serves to clear waste products.

Through the NVU, neurons are able to communicate to their local vasculature directly as well as indirectly. In the first instance, activated neurons can directly target vessels, typically larger vessels such as arterioles, through the release of factors such as nitric oxide and prostaglandin E_2_ to induce vasodilation in vascular smooth muscle cells^35,45^. Alternatively, neurons can signal through the NVU whereby neuronal-glia communication occurs via the release of neurotransmitters, with a calcium-dependent glial release of vasodilatory agents responsible for modification of vessel caliber^35,45,46^. Several studies have investigated this indirect signaling pathway within the retina and the Müller cell, a macroglial cell specific to the retina, has been shown to form a key component of the retinal NVU, with light-evoked increases in Ca^2+^ activity associated with changes in vessel caliber^47,48^. More specifically, recent work reveals that Müller cells are exclusively involved in regulating the intermediate vascular plexus, leaving other cell types / mechanisms to be involved in the regulation of the remaining retinal plexuses (SVP and DVP)^48^.

**Fig. 3:**
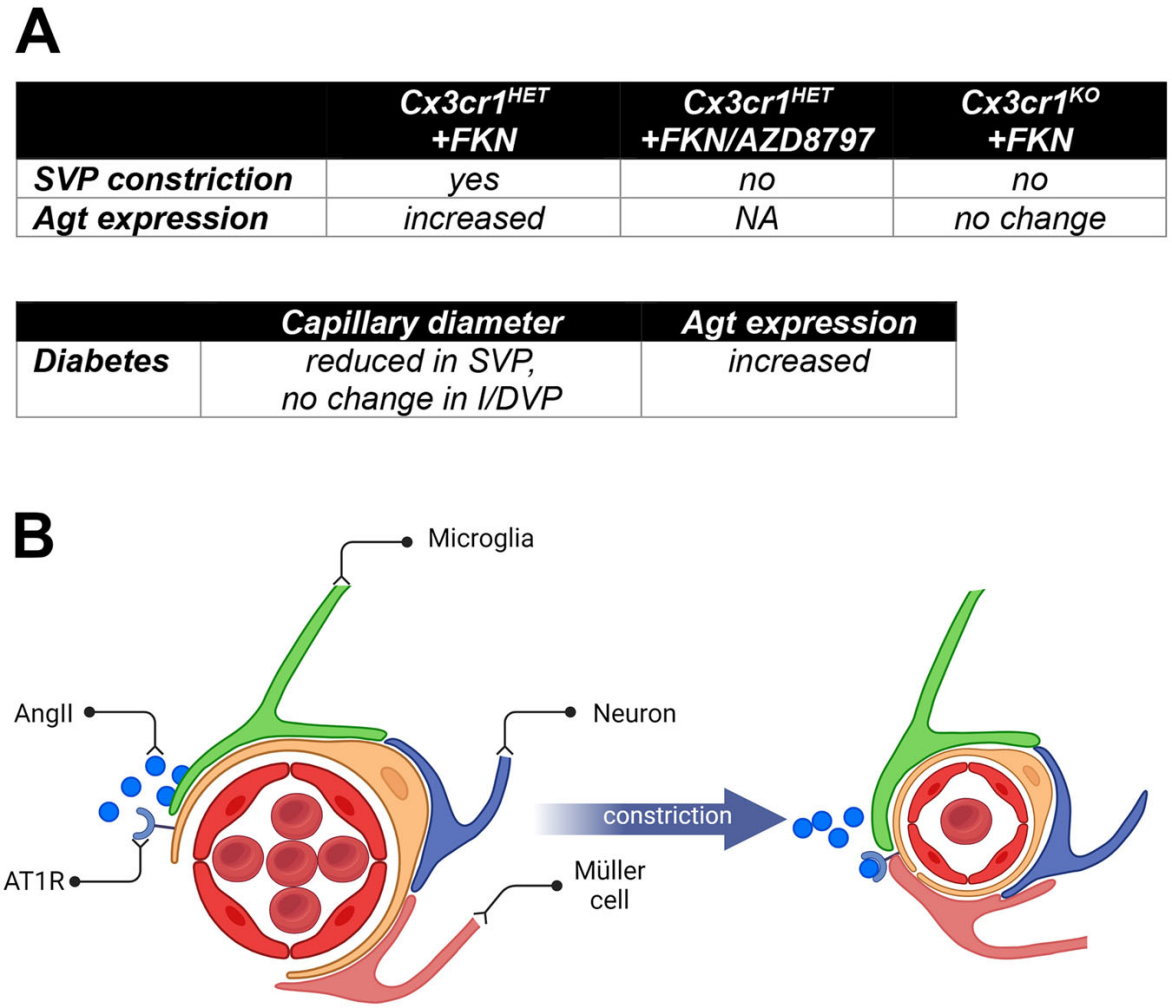
The mechanism by which fractalkine induces constriction of blood vessels and how it is modified by diabetes. **A** A summary of data taken from Mills et al.^12^ detailing fractalkine-dependent microglial vasoregulation within the retina and how it is altered during diabetes. **B** A schematic of the proposed microglial-mediated vasoconstriction via the renin-angiotensin system. Capillaries are contacted by processes of Müller cells, microglia and neurons. Fractalkine released from neurons induces the release of mediators of the renin-angiotensin system from microglia, including angiotensin II, which binds to AT1R receptors expressed by pericytes, causing their contraction. Created with Biorender.com.

Microglial involvement in NVC has been suggested, given that these cells exhibit structural and functional interactions with both neurons, vasculature, and Müller cells (see Fig. 1B, C)^12,21,49^. Retinal microglia within the SVP are known to make concurrent contact with capillaries and excitatory synaptic terminals (vesicular glutamate transporter 1 positive synapses) in the inner retina of both the mouse and human retina^12^. In addition, live cell imaging of retinal explants revealed that microglia were associated with vasoconstriction of capillaries, an effect that was dependent on Cx3cl1 (fractalkine)/Cx3cr1 signaling, a mechanism by which neurons communicate with microglia^50,51^. Indeed, fractalkine induced microglial-mediated vasoconstriction was abolished in retinal explants from Cx3cr1^GFP/GFP^ mice, as well as after the pre-incubation with the Cx3cr1 inhibitor AZD8797^12^. Based on functional as well as molecular analyses, components of the renin-angiotensin system, potent regulators of the vasculature, were identified as mediating the communication between retinal microglia and inner retinal blood vessels (see Fig. 3)^12^. Taken together, it is possible that there are multiple mechanisms by which retinal capillaries are regulated in response to neural needs – one involving Müller cells and another involving microglia (Fig. 3).

## Early vascular changes in the diabetic retina and the involvement of microglia

The demanding metabolic nature of the retina renders it vulnerable to metabolic insults such as ischemia or diabetes. One of the earliest pre-clinical changes that occurs in the diabetic retina, is a reduction in blood flow. Highlighting this, retinal blood flow was reduced by ~34% in diabetic individuals in the absence of visible DR^8^. Similar findings have been replicated using a variety of techniques in experimental models^52–58^. In addition, alterations in the basal diameter of larger vessels are a common feature in the diabetic eye. For example, mean arteriole diameter is reduced in individuals with type 1 diabetes and found to gradually decrease, with increasing severity of DR, by 3.23 μm per level on the retinopathy grading scale^9,59^.

Evidence from clinical studies and animal model work suggests that these vascular anomalies are attributed to dysfunction of the underlying mechanisms governing vasoregulation. In line with this, numerous studies demonstrate that the retinal microvasculature in diabetic individuals exhibits diminished functional hyperaemic responses, indicative of impaired NVC^10,60–66^. In fact, retinal responses to photopic stimulation are found to worsen with increasing DR severity, as controls exhibit a robust 3.6% light-evoked increase in arteriole diameter, compared to diabetic individuals with no DR, mild non-proliferative DR (NPDR), moderate NPDR, severe NPDR and proliferative DR (PDR), each exhibiting markedly reduced responses of 2.6%, 2.0%, 1.6%, 1.8% and 0.8%, respectively^65^. Similar findings of altered vessel response are also observed in experimental models^67,68^. These findings strongly implicate early vascular dysfunction as a key driver in the development of DR. Given the high metabolic requirements of the retina and its almost negligible capacity to store energy, the resultant ischemia from even minor disruptions in blood flow in diabetes can have severe consequences on the functioning and survival of retinal neurons. Moreover, retinal ischemia is a well-established contributing factor to the development of more advanced vascular pathology such as neovascularization and vascular leakage, by promoting the upregulation of pro-angiogenic factors^4^.

In exploring the mechanism behind such diabetes-induced dysfunction in vasoregulatory control, studies have shown that upregulation of inducible nitric oxide synthase (iNOS) reduces light-evoked vessel dilation likely impacting the expression of vasoactive factors within Müller cells^46^. Indeed, there is a wealth of information highlighting that Müller cells are altered by diabetes^69^, and that blockage of iNOS with amioguanidine can abrogate the loss of vascular function^43,67^. Similarly, studies have also shown that diabetes induced changes in vascular endothelial cells and pericytes may also impact on normal vascular control^70^.

Similarly, the vasoregulatory function of microglia may be compromised at an early stage of and associated with vascular impairment. Indeed, basal blood flow is reduced in the 4-week streptozotocin (STZ) rat, an effect that was associated with sustained vasoconstriction^12^. In addition, inner retinal blood vessels failed to change calibre in response to the application of the vasoconstrictor, fractalkine, or exposure to high oxygen. These vascular changes were observed in the absence of any signs of microglial inflammation, vessel loss, neurodegeneration, or gliosis, while there were increases noted in the association of microglia with capillaries in the SVP, and increased retinal fractalkine expression. However, expression of the RAS-related gene angiotensinogen (*Agt*) was 2.4-fold higher in microglia isolated from STZ rat retinae compared to non-diabetic controls, implicating the microglial RAS in the aberrant vasoconstriction and poor vascular reactivity observed in early diabetes^12^.

Although microglia are implicated in early vascular compromise, it remains to be examined whether vasoregulation is a function also shared by deeper microglia in the retina associated with deeper capillary beds within the IVP and DVP. Moreover, the role of microglial regulation of arterioles and venules remains to be investigated. Importantly, whilst SVP-associated microglia appear to be principally involved in early vascular compromise in diabetes, uncovering the vasoregulatory capacity of all microglia in the healthy retina, will assist in a deeper understanding of the exact contribution of all microglia during DR.

## Microglia as prominent contributors to retinal inflammation in late-stage DR

As the resident immune cells of the retina, microglial changes in the diabetic eye have historically centered around their inflammatory-dependent roles in the later stages of DR. Early on in the diabetic retina, the initial response of microglia is neuroprotective and anti-inflammatory by releasing cytokines to help alleviate retinal stress and promote neuronal survival^71^. However, prolonged tissue stress caused by the chronic nature of diabetes, promotes microglia to shift into a progressively more pro-inflammatory and neurotoxic state, significantly contributing to chronic inflammation.

Indeed, overwhelming evidence supports that microglia undergo a variety of structural and functional changes indicative of a shift from homeostatic to pro-inflammatory phenotype. Illustrating this, microglia in retinae from individuals with NPDR and PDR are observed to exhibit an activated phenotype, reflected by the adoption of a more ameboid shape, and increased cell density indicative of proliferation and migration into spaces in which they normally do not reside, such as in the ONL and subretinal space^72^. These findings are also observed in experimental models of diabetes and there are significant alternations in the transcriptomic signature of microglia, reflected by downregulation in homeostatic genes and upregulation in those predominately associated with pro-inflammatory signaling, chemotaxis and proliferation^73–75^. In response to diabetes, microglia upregulate the expression of pro-inflammatory cytokines including tumour necrosis factor-alpha (TNF-α) and interleukin-1 beta (IL-1β), as well as cytotoxic molecules such as reactive oxygen species (ROS)^71,76,77^. Whilst other cells can contribute to the production of these factors, transcriptomic analysis in 4-month diabetic mice found microglia to be the major sources of IL-1β and TNF production^78^.

Directly implicating microglia in the pathology associated with DR, microglial ablation in diabetic rodents prevented retinal dysfunction and retinal thinning^75^. In another study, microglial depletion was conducted after several weeks of diabetes followed by a recovery period to allow for microglial repopulation^79^. Assessment of the new population of microglia found that these cells exhibited a less activated morphology and had a neuroprotective affect evidenced by attenuated axonal loss, suggesting that these microglia were less inflammatory and neurotoxic. This was further supported by transcriptomic analysis of the repopulated microglia revealing a downregulation in genes associated with microglial activation, whilst genes that would confer neuroprotection were conversely upregulated^79^.

## The therapeutic potential of targeting microglia in DR

**Fig. 4:**
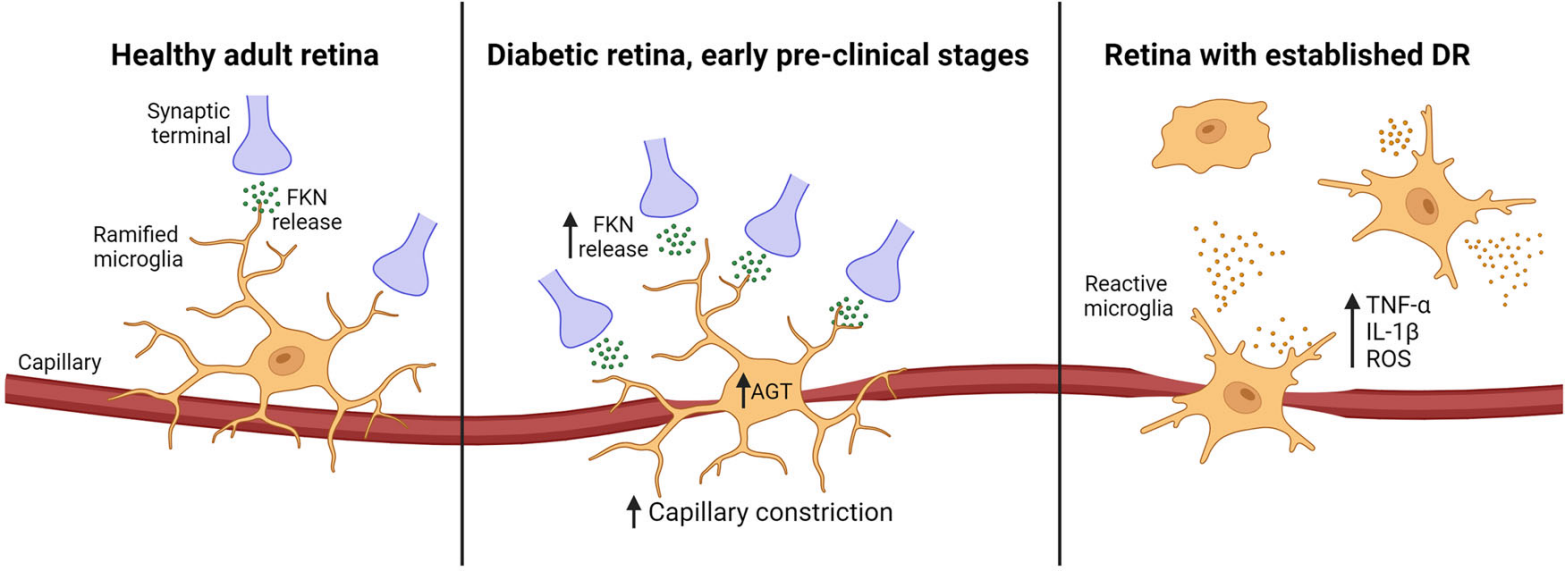
Schematic diagram of the changes in vascular regulation that are predicted to occur at different stages of diabetes. In the healthy mature retina, microglia play a variety of roles critical in maintaining tissue homeostasis. This includes vasoregulation, where neurons signal to microglia via fractalkine (FKN), which stimulates the RAS-dependent constriction of capillaries. Early in the diabetic eye, upregulated fractalkine expression in the retina results in increased microglial-induced vasoconstriction, reflected by increased microglial-capillary contact and angiotensinogen (Agt) expression. This is strongly associated with capillary narrowing and reduced retinal blood flow. These vascular anomalies create an ischemic environment and given the high energy demand of the retina, this has severe consequences on overall retinal function and survival. Due to the chronic nature of diabetes, prolonged exposure of microglia to tissue stress eventually triggers these cells to become reactive, shifting into a more pro-inflammatory state denoted by morphological and molecular changes. Through the upregulation of pro-inflammatory cytokines including tumour necrosis factor-alpha (TNF-α) and interleukin-1 beta (IL-1β), as well as cytotoxic molecules such as reactive oxygen species (ROS), reactive microglia are highly neurotoxic. This further compromises neuronal survival, exacerbating DR pathology. Created with BioRender.com.

Current treatment options for later-stage DR are effective in preserving vision. These include laser photocoagulation, intravitreal injections of anti-vascular endothelial growth factor (VEGF), and vitreoretinal surgery, all of which help to reduce macular oedema and neovascularization^4^. Nevertheless, these treatments are tailored exclusively for the advanced stages of the disease and do not halt progression, nor reverse vision loss. Hence, there is a need to discover alternative therapeutic strategies for the management of DR, for early intervention to prevent disease onset and delay progression. As depicted by Fig. 4, microglia are implicated in the early and late stages of the disease, rendering these cells attractive therapeutic targets to prevent or mitigate vasoregulatory dysfunction and inflammation, which are key components of DR development.

With regards to inflammation, several lines of evidence demonstrate therapeutic benefit in inhibiting microglial activation. Anti-inflammatory treatments, including minocycline or baicalein, attenuate the upregulation of IL-1β and TNF-α and confer retinal protection in animal models of diabetes as demonstrated by reductions in neuronal loss, capillary loss, and vascular leakage^77,80^. Furthermore, minocycline treatment in a pilot study of individuals with diabetic macular oedema, was associated with modest improvements in visual acuity, macular oedema, and vascular leakage^81^. Doxycycline treatment in individuals with advanced DR, including severe NPDR and PDR, also exhibited improved inner retinal function^82^. While these treatments may hold promise for later-stage disease, doxycycline conferred no benefit in those with earlier stages of DR, suggesting that the drug may be more effective in later stages of disease when inflammation is more severe^82^. Whilst these preliminary clinical findings are promising, subsequent studies with larger sample sizes are required to clarify the benefit of these treatments.

The use of traditional corticosteroid treatments such as triamcinolone acetonide (TA), have also been investigated in DR due to their anti-inflammatory and anti-angiogenic properties. For individuals with diabetic macular oedema that are resistant to conventional treatment, intravitreal injections of TA have been shown to confer great benefit, by reducing oedema and improving visual outcomes^83,84^. Nonetheless, the usage of this treatment remains limited based on its associated side effects, including cataract and increased intraocular pressure which can lead to glaucoma^85^. In order to improve drug efficacy whilst reducing the risk of adverse effects, recent work has assessed the use of dendrimer nanoparticles for targeted delivery to retinal microglia. Dendrimers are observed to selectively target activated microglia in several ocular pathologies, including in a murine model of oxygen-induced retinopathy (OIR), as well as laser-induced glaucoma^86,87^. Such delivery of TA was shown to reduce retinal expression of pro-inflammatory genes, reduce pathological neovascularization, and improve retinal function in the mouse model of OIR. These findings are incredibly promising and highlight the efficacy of dendrimers, as a novel delivery system for TA to selectively target and suppress activated microglia. Whilst the efficacy of dendrimer TA treatment is yet to be directly examined in experimental models of DR, the OIR model is very useful in studying the angiogenic component of DR indirectly. Further testing will thus clarify the potential of this treatment as an intervention for DR.

Other studies have targeted inflammation through the use of monoclonal antibodies against the pro-inflammatory cytokine TNF-α (Infliximab), with it shown to be effective in improving vision in diabetic individuals with diabetic macular oedema^88,89^. However, further investigation into the optimal treatment regime for this drug is required, to help limit the rare, yet severe, adverse effects with which it is associated^90^. Similarly, recent work has also uncovered the therapeutic potential of targeting the CD200-CD200R signaling axis between neurons and microglia in order to limit inflammation. Typically, CD200 is released by neurons and binds to its receptor which is expressed chiefly by myeloid-derived cells (including microglia), and functions to dampen immune responses^91^. In diabetic mice, CD200-CD200R signaling was markedly attenuated and CD200 fusion protein (CD200Fc) treatment used to enhance this signaling pathway was found to prevent microglial activation, retinal inflammation, and importantly, retinal dysfunction^75^. While CD200-CD200R signaling is a promising therapeutic target, further studies assessing the more long-term effects of CD200Fc treatment will help clarify its immunotherapeutic potential.

While interventions that prevent or mitigate microglial vasoregulatory dysfunction may also prove effective in DR, currently there are few studies exploring such treatments. Given the involvement of increased fractalkine expression in retinal vasoconstriction during diabetes, inhibition of this chemokine may be therapeutically beneficial. However, the key role of fractalkine signaling in controlling normal microglial surveillance renders this challenging^24^. Demonstrating this, studies show that the ablation of fractalkine signaling results in increased neurodegeneration, number of acellular capillaries, microglial activation and macrophage accumulation in the retina in diabetic mice (Cx3cr1null)^92–94^. Conversely, intravitreal injection of fractalkine in diabetic rodents has been shown to reduce microglial activation and retinal inflammation^95^. Therefore, the apparent concentration-dependent nature of fractalkine in the diabetic and normal retina complicates the therapeutic potential of this type of treatment. One alternative treatment option would be to target the increased microglial RAS activity, such as through AT1R inhibition. Indeed, the AT1R antagonist losartan is known to reduce the risk of DR progression in diabetic individuals^96^, while candesartan treatment also reduced disease incidence, but had no impact on disease progression^97^. Similarly, candesartan treatment in a rodent model of diabetes alleviated the retinal capillary constriction, however, did not resolve the reduced retinal blood flow due to “off target’ effects on the larger retinal vessels^12^. Additional work is thus required to help develop new treatments specifically targeted at the interaction between microglia and retinal capillaries in order to restore retinal blood flow and preserve retinal function.

## Conclusions

In summary, diabetic retinopathy remains one of the leading causes of visual impairment. It is now widely accepted that it is more than just a vascular disease denoted by visible vascular pathology, as diabetes is currently understood to affect the retina globally, impacting essentially all the cells with which it is comprised. The contribution of microglia, the resident immune cells of the CNS, has historically centered around their inflammatory functions in DR. Through various approaches including microglial ablation, targeting microglial-specific signaling pathways, and suppressing microglial activation, work has investigated the role of microglia in DR and cemented these cells as keys players in disease development through inflammation. However, beyond this, recent work has additionally uncovered a novel role of retinal microglia as functional contributors in neurovascular coupling enabling precise control of retinal vessel tone. Subtle changes in the regulation of blood flow, are one of the earliest pre-clinical changes observed in the diabetic eye, indicative of aberrant neurovascular coupling. Furthermore, experimental work reveals microglial-mediated vasoregulation to become dysfunctional in diabetes, which is strongly associated with early vascular impairment including capillary narrowing and reduced blood flow. These findings highlight the contribution of microglia from the pre-clinical to the clinical stages of DR and make microglia an attractive therapeutic target, to address both vascular and inflammatory components of DR.
